# Establishment of a Radiation Dose–Response Calibration Curve Using a Rapid Cytokinesis-block Micronucleus Assay for Dose Assessment of Medical Radiation Equipment

**DOI:** 10.14293/genint.15.1.003

**Published:** 2025-05-14

**Authors:** Ji Young Kim, Seong-Jun Cho, Hoon Choi, Jeongin Kim, Il Hyeok Seo, Se Hyun Kim, Mooyoung Yoo

**Affiliations:** 1 Radiation Health Institute, Korea Hydro & Nuclear Power Co. Ltd, Seoul, Republic of Korea; 2 A-BNCT Center, Dawonmedax, Incheon, Republic of Korea

**Keywords:** Dose–response calibration curve, cytokinesis-block micronucleus assay, radiation, CHO-K1 cells

## Abstract

Dicentric chromosome analysis (DCA) has limitations in its use for the evaluation of the radiation dose upon the development of medical radiation equipment due to its time/labour-consuming procedure and the requirement of highly trained experts. Therefore, we aimed to construct a dose–response curve using a semi-automatic cytokinesis-block micronucleus (CBMN) analysis method that can be easily analysed and utilised by anyone. CHO-K1 cells were exposed to gamma rays at various doses (0–4 Gy). For the CBMN assay, the bi-nucleated cells were selected and captured, and micronuclei (MN) scoring was automatically performed using the Metafer4 system. The MN scores were manually confirmed and corrected by analysts. Using the frequency distributions of MN according to the radiation dose, the dose–response calibration curve was generated using Dose Estimate v5.2 software. The equation of dose–response calibration curve is Y = 0.0299 (±0.0057) + 0.1502 (±0.0151) × D + 0.0111 (±0.0048) × D^2^. The goodness-of-fit parameters were also calculated (chi-squared [*χ* ^2^] = 39.45, degrees of freedom = 5, *p* = 0.0000). The semi-automated CBMN assay consist of two steps: the automated MN capture/scoring step and the manual confirmation/correction step. Using an established dose–response calibration curve and the procedure of the semi-automated CBMN assay, the dose-estimation of gamma-irradiated (0.5 or 2 Gy) CHO-K1 cells were performed by two analysts individually, and it was inter-compared to verify the accuracy, the results showed that the estimated doses were a good fit the applied doses of radiation. The CBMN assay using CHO-K1 cells can be easily used as a biodosimetry tool for dose assessment of medical radiation equipment due to the advantage of being simple, easy, and quick to measure the dose.

## Introduction

Currently, radiation is widely utilised in various fields such as industry, medical diagnosis, and therapy, etc. Especially, the radiation therapy market is a rapidly growing field in the healthcare industry, driven mainly by the increasing prevalence of cancer and the development of radiation therapy technology. According to data released in the USA in January 2024, the radiation therapy market is expected to grow by $1.59 billion from 2023 to 2028. As the radiation therapy market grows, the demand for the development of radiation therapy equipment will be increased, and biological dose evaluation as well as physical dose evaluation for radiation devices will be necessary for the development this equipment.

Based on the recommendations of the International Atomic Energy Agency, various tools of biological dosimetry such as dicentric chromosome analysis (DCA), translocation (TR) analysis, premature chromosome condensation analysis and cytokinesis-block micronucleus (CBMN) analysis, were presented to accurately and promptly evaluate the individual absorbed dose.^[1]^ Various biodosimetry tools are utilised mostly on urgent radiation dose evaluation for emergency situations or retrospective studies. Among them, DCA is commonly considered the “gold standard” technique of biodosimetry.^[2]^ Besides its use in radiation emergencies, biodosimetry is being utilised for the evaluation of biological effectiveness for novel types of medical radiation equipment such as in boron neutron capture reactors and alpha-particle irradiators.^[3,4]^ Although DCA is considered the “gold standard” technique, it still has limitations in being easily utilised as a biodosimetry tool. For instance, DCA still requires time/labour-consuming procedures and highly trained experts who have experience with DC scoring. For that reason, the biodosimetry tools which are faster and easier than DCA to use are currently required.

Here, we established a dose calibration curve for CBMN assay in gamma-irradiated CHO-K1 cells. To shorten the time of analysis, we utilised an automated image capture system using Metafer4 equipment that allows the specific selection of images of bi-nucleated cells. The CBMN assay procedure applying an automated image capture system will be a useful tool to evaluate the biological effectiveness of medical radiation equipment.

## Materials and Methods

### Cell culture and irradiation

The CHO-K1 cell line was obtained from the Korea Cell Line Bank (KCLB; Seoul, Korea). CHO-K1 cells were cultured in RPMI-1640 (Cytiva, Marlborough, MA, USA) with 100 units/mL penicillin-streptomycin (Gibco, Norristown, PA, USA), and 10% (v/v) foetal bovine serum (Gibco) at 37°C under a 5% carbon dioxide (CO_2_) atmosphere. A total of 2 × 10^5^ CHO-K1 cells were seeded into 35 mm dish (SPL, Gyeonggi-do, Korea) with culture medium at 37°C, with 5% CO_2_ for 24 h. Cell dishes were then placed in Gamma-cell^®^ 40 Exactor of ^137^Cs source (Best Theratronics, Ottawa, Canada) and irradiated with doses of 0, 0.25, 0.5, 0.75, 1, 2, 3, and 4 Gy (0.8 Gy/min), respectively.

### Slide preparation for bi-nucleated cell detection

The irradiated cells were incubated at 37°C, 5% CO_2_ for 4 h, then treated with 3 μg/mL of cytochalasin B (Merck, Germany) and incubated for 20 h. The medium was removed, and cells were harvested into 15 mL tubes using Trypsin-EDTA (Gibco). Cells were harvested by centrifugation and washed once with phosphate-buffered saline (PBS; pH7.4). Cells were then resuspended in Fixative 1 solution (45.7%, methanol, 8.3% acetic acid, 0.45% NaCl) using a vortex mixer and placed on ice for 10 min. Cells were centrifuged and resuspended in Fixative 2 solution (83.3% methanol, 16.7% acetic acid) using a vortex mixer and placed on ice for 10 min. The cell fixation step with the Fixative 2 solution was repeated four times. Finally, cell pellets were resuspended in Fixative 2 solution and transferred into microtubes and stored at 4°C for more than 8 h. For slide preparation, cells were centrifuged and resuspended in Fixative 2 solution using a vortex mixer. Cell suspension was dropped and spread onto a slide glass (VWR, Radnor, PA, USA) and allowed to air dry. To stain the nucleus with 4’,6-diamidino-2-phenylindole (DAPI), the mounting medium with DAPI solution (Abcam, UK) was dropped onto a slide glass, and covered with a cover glass (MARIENFELD, Germany). Slides were stored at −20°C for more than 8 h.

### Capture of bi-nucleated cell image and micronuclei scoring

The micronuclei (MN) scores, initially determined using the Metafer4 system, were manually confirmed and corrected by analysts to address errors such as off-focused cells, mononuclear cells, and misclassified MN. The images of the DAPI-stained/bi-nucleated cells were obtained using the Metafer4 system (Metasystem, Heidelberg, Germany) as previously described.^[5]^ Frequencies of MN were measured in more than 4,000 bi-nucleated cells for every radiation dose. Also, the accuracy of the dose evaluation was compared and analysed by allowing two analysts to conduct dose analysis on samples irradiated with 0.5 and 2 Gy of gamma rays according to the procedure.

### Data analysis

A dose–response calibration curve was constructed using Dose Estimate software (version 5.2) using the frequencies of MN according to the radiation dose, and the dose for the irradiated sample was evaluated using this dose curve.

## Results

**Figure 1:**
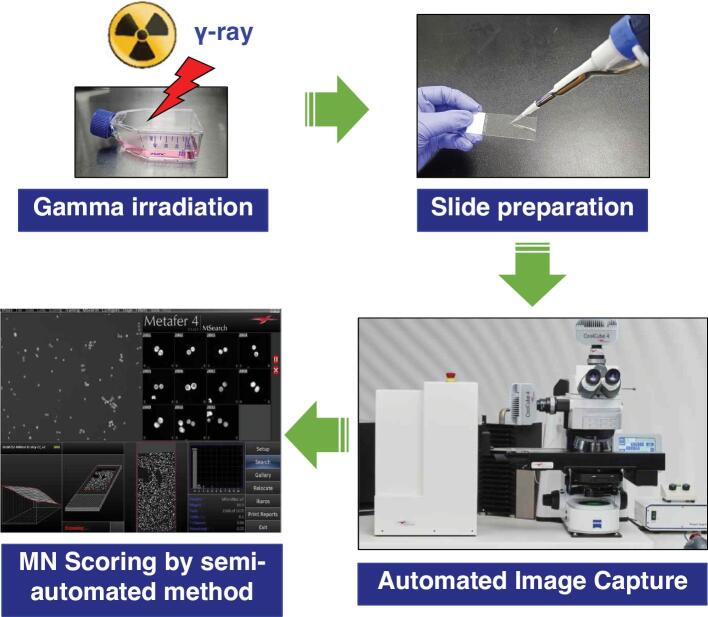
Experimental procedure for a CBMN assay using semi-automated MN scoring.

**Figure 2:**
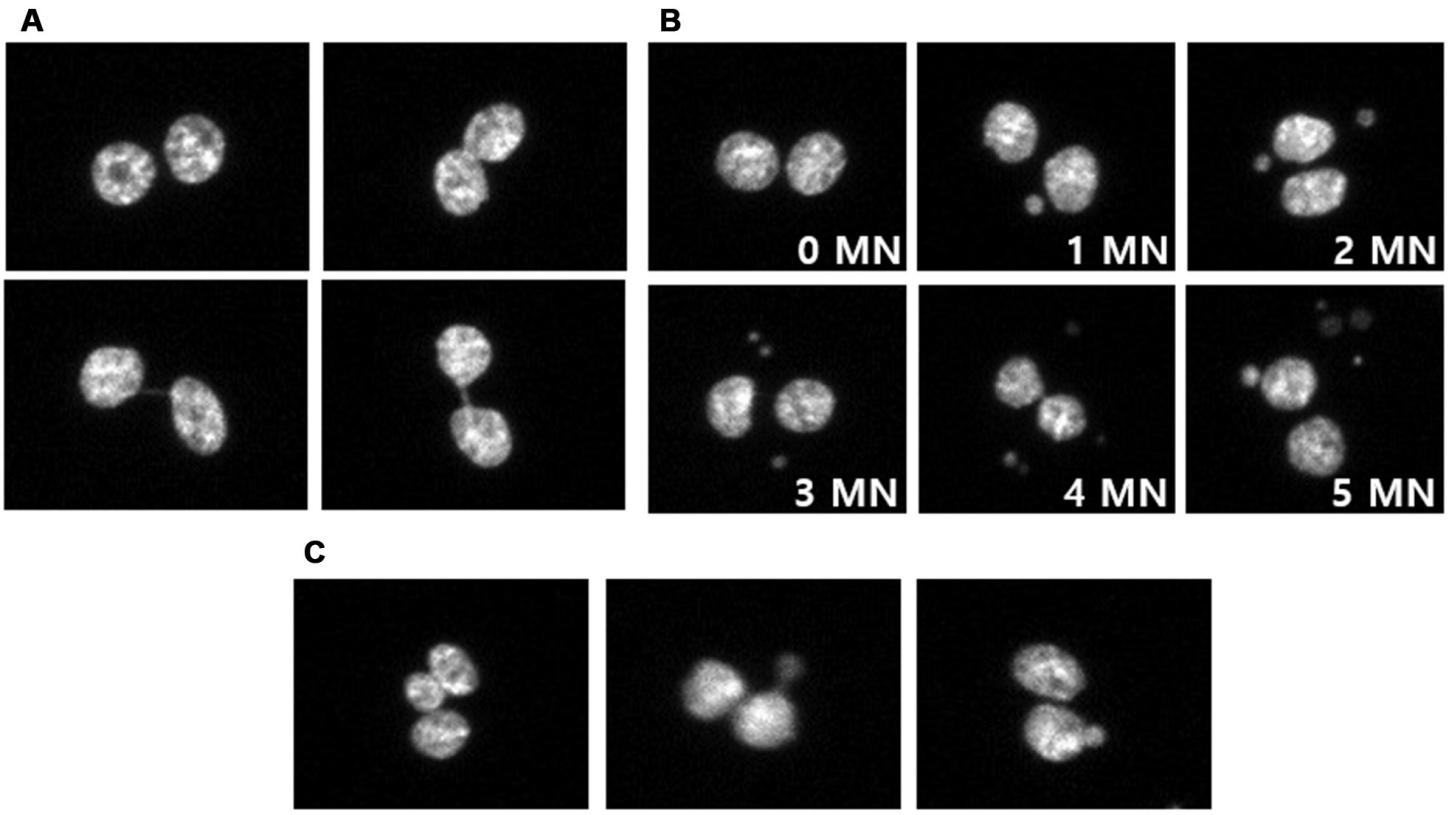
Representative images of selected bi-nucleated cells (A) containing various numbers of MN (B) or discarded cells (C).

To establish the dose–response calibration curve for the CBMN assay, we performed cultivation of the CHO-K1 cells, irradiation, slide preparation, and MN scoring as described in the Materials and Methods section and shown in Figure 1. The analysts selected more than 4,000 bi-nucleated cells from captured images using the Metafer4 system. The Metafer4 system can automatically capture the bi-nucleated cells and roughly classify the micronucleated cells as the number of micronuclei. However, selected bi-nucleated cells and classified micronucleated cells using the Metafer4 system were incomplete, and it needed additional manual correction. For example, images of the selected bi-nucleated cells still contained off-focused cells, mononuclear cells and smaller bi-nucleated cells which needed to be discarded. The representative images of selected types for bi-nucleated or micronucleated cells are shown in Figure 2A and B, respectively. In the classified micronucleated cell images, some of images were still miss-classified as the number of MN or did not fit the selection criteria of MN. Thus, the analyst corrected the MN images manually. The verification of the MN score was performed as per the criteria described previously.^[6]^ Briefly, we discarded tri-nucleated cell images that contained the smallest nuclei, which were greater than 1/3 diameter of the other nuclei, and the images containing extruded nuclear material resembling micronucleus with a nucleoplasmic coupling to the major nucleus or nuclear blebs that have an obvious nucleoplasmic coupling with the major nucleus (Figure 2C).^[6]^ The frequency distributions of MN in CHO-K1 cells which were exposed to several doses of gamma rays are shown in Table 1.

**Table 1:** Frequency distributions of MN in CHO-K1 cell exposed to gamma rays.

Dose (Gy)	Cells	Aberrations	The number of MN per cell					
			0	1	2	3	4	>5
0	6,812	212	6,623	169	17	3	0	0
0.25	8,233	558	7,719	474	36	4	0	0
0.5	5,673	567	5,159	465	46	2	1	0
0.75	7,979	1,136	6,972	890	105	12	0	0
1	6,621	1,392	5,411	1,050	139	20	1	0
2	5,963	2,095	4,293	1,322	280	59	9	0
3	4,129	2,573	2,214	1,391	417	84	19	4
4	4,470	3,522	2,050	1,585	626	163	34	12

**Figure 3:**
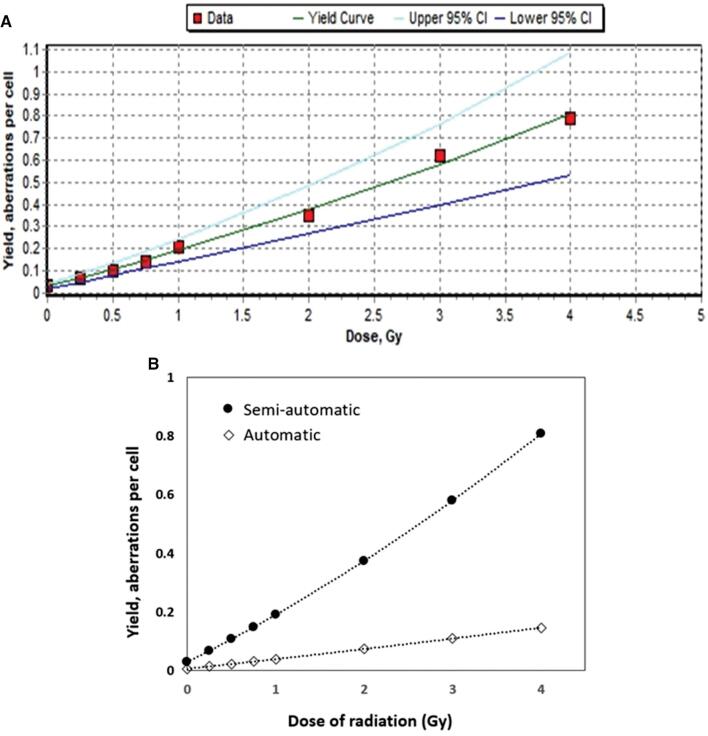
Linear-quadratic dose–response calibration curve for a CBMN assay with gamma-irradiated CHO-K1 cells using Dose Estimate v5.2 software. (A) Established dose–response calibration curve for a semi-automated CBMN assay and its corresponding 95% upper/lower confidence limits. (B) Comparison of dose–response calibration curves for semi-automated and automated CBMN assays.

The dose–response calibration curve of MN is usually best fitted by a linear-quadratic model:

$$\text{Y}=\text{c}(\pm \text{SE})\;+\;\alpha (\pm \text{SE})\text{D}\;+\;\beta (\pm \text{SE}){\text{D}}^{2},$$

where Y is the number of MN in cells; D is the radiation dose (Gy); and c, α and β are the unknown parameters of the model. Using the Dose Estimate v5.2, the dose–response calibration curve was generated (Figure 3A) and the equation is:

$$\begin{array}{c} \text{Y}=0.0299\;(\pm 0.0057)\;+\;0.1502\;(\pm 0.0151)\\ \;\times \;\text{D}+0.0111\;(\pm 0.0048)\;\times \;{\text{D}}^{2}. \end{array}$$

The corresponding 95% upper/lower confidence limits are also shown in Figure 3A. The goodness-of-fit parameters were also calculated by the weighted chi-squared [*χ* ^2^] test provided by Dose Estimate V5.2 software (*χ* ^2^ = 39.45, Degrees of Freedom = 5, *p* = 0.0000). The correlation coefficients were highly close to 1 (r = 0.997), suggesting a strong relationship between the fitted data points.

In addition, we also generated the dose–response calibration curve for automated MN assay to compare its accuracy with that of semi-automated MN assay. And the equation is:

Y=0.0069 (±0.0022) + 0.0322 (±0.0058) × D + 0.0007 (±0.0018) × D2.

The goodness-of-fit parameters were also calculated by the weighted *χ* ^2^ test provided by the Dose Estimate V5.2 software (*χ* ^2^ = 30.47, degrees of freedom = 5, *p* = 0.0000). The correlation coefficient (r) was 0.9916. As shown in Figure 3B, the slope of the dose–response calibration curve for the automated MN assay was much lower than that of semi-automated MN assay due to its low detection of MN.

To evaluate the accuracy of our semi-automated CBMN assay and ease of the evaluation method, two analysts with no experience using the CBMN assay, but who had some knowledge of the MN selection criteria, assessed the doses for CHO-K1 cells irradiation with 0.5, 2 Gy radiation using a dose–response calibration curve according to the procedure. The MN scores measured by the two analysts were applied to the established dose–response calibration curve and the estimated doses were determined using Dose Estimate v5.2 software and the estimated doses were compared with that of the automated method applied with the dose–response calibration curve for the automated MN assay.

**Figure 4:**
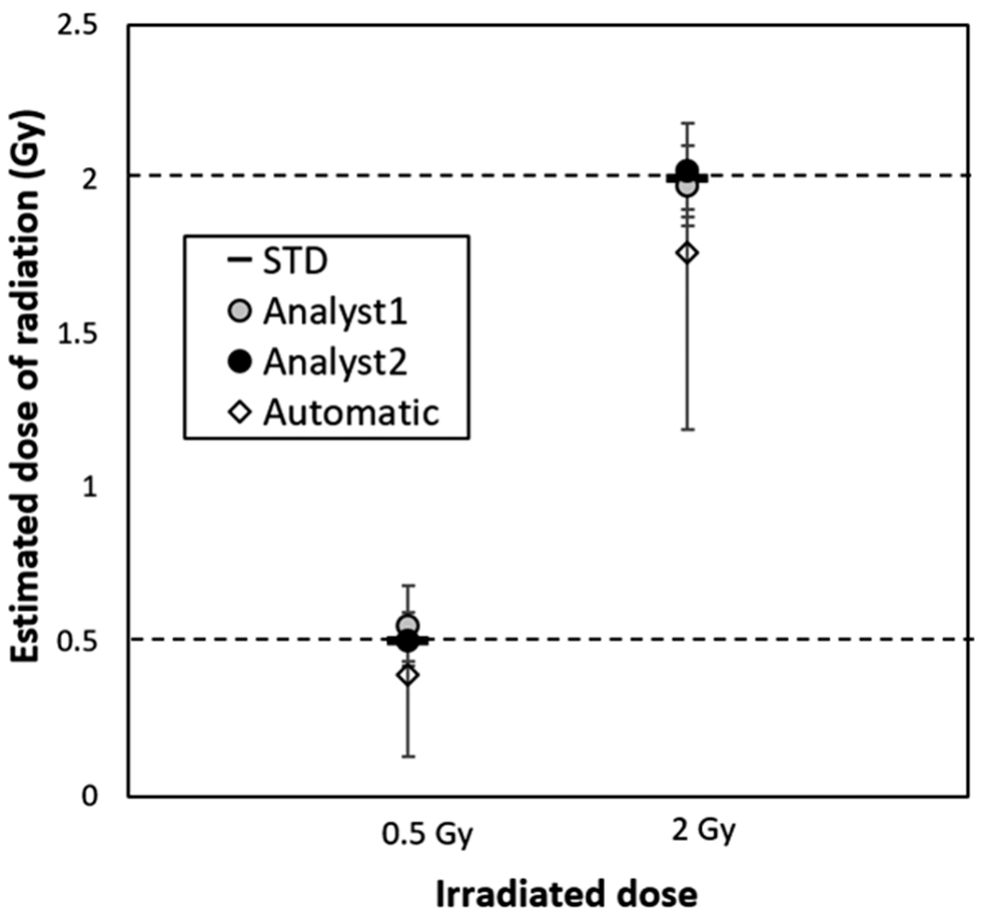
Dose estimation with MN scores in gamma-irradiated CHO-K1 cells to verify the accuracy of constructed a dose–response calibration curve. The samples from CHO-K1 cells exposed to 0.5 or 2 Gy of radiation doses were prepared by two analysts and the CBMN assays were performed, respectively. The estimated doses by two analysts were compared with doses from automated dose estimation using the Metafer system. Upper limits and lower limits of 95% confidence for each sample were indicated. STD: standard dose.

Our data showed that estimated doses measured by two different analysts were almost consistent with their applied doses at 0.5 or 2 Gy of radiation (Figure 4). The estimated doses were 0.56 and 0.51 Gy for the applied dose at 0.5 Gy, and 1.98 and 2.03 Gy for 2 Gy, respectively. Unlike with this method, the estimated doses (0.40 Gy for applied dose at 0.5 Gy, and 1.76 Gy for 2 Gy) measured using the automatic procedure was much lower than the applied doses, indicating that our semi-automated method substantially improved the accuracy of dose estimation. Moreover, our data also showed that each estimate dose was within the 95% upper/lower confidence limit range, suggesting that the semi-automated CBMN assay and dose–response curve is highly accurate and easy to analyse.

## Discussion

So far, numerous biodosimetry tools have been developed and are being utilised in various radiological fields such as radiation emergency preparedness, retrospective studies for epidemiology and the development of novel radiation equipment for therapy.^[3,4,7–9]^ However, due to the high level requirements for and limitation of biodosimetry such as needing highly trained experts and the labour/time-consuming process, it is not easy to utilise in various radiological situations. As regards the use of biodosimetry, the CBMN assay is a comparatively easier tool to use than other biodosimetry tools because this tool does not require highly trained experts for MN scoring. However, the CBMN assay also takes a lot of time to measure, particularly, as regards cell culture and MN scoring. Thus, to improve the CBMN assay, we applied an automated selection/scoring system to speed up the process, and a manual correction step to compensate for the incomplete scoring by the automated system and improve its accuracy. Similar to our study, a previous report showed that a semi-automated CBMN assay with human blood lymphocyte substantially improved its speed, accuracy, and efficiency compared with the fully automated or manual CBMN assay.^[10]^ These results demonstrate that a semi-automated CBMN assay provides advantages for dose-estimation in urgent situations.

In the experiment to establish a dose–response calibration curve, most analysts used human peripheral lymphocytes.^[11,12]^ Because blood cells, particularly blood lymphocytes, are the most sensitive tissue type against radiation exposure, it is considered as the most reliable substrate to use in biodosimetry.

Even though using human blood is invasive and difficult due to several obstacles such as ethics, it is best to use the blood cells from a patient exposed to radiation for dose assessment in the event of a radiation accident and related research. Whereas there is no need to use human blood cells for dose estimation for the performance of radiation medical devices. Therefore, in our experiment, we used CHO-K1 cells to establish the semi-automated CBMN assay and its dose–response calibration curve. Previously, the CBMN assay in CHO-K1 cells has been used as a biological dosimeter to measure the effectiveness of medical devices such as: small animal irradiator, a device used for radiating small lab animals in research.^[13]^ Additionally, our CBMN assay in CHO-K1 cells could be useful for the development of a radiation sensitiser. For instance, boron neutron capture therapy is a method of injecting boron into a cancer patient and the irradiating neutron to increase the efficacy of radiotherapy. At this case, if our CBMN assay in CHO-K1 cells is applied, it will be a way to easily verify detailed factors such as the appropriate amount of boron and processing time *in vitro*.

The use of CHO-K1 cells for biological dosimeter provides several advantages in terms of non-invasiveness and non-ethical issue. Thus, our method of using a semi-automated CBMN assay with CHO-K1 cells and its dose–response calibration curve can be used as an easier, rapid, accurate and efficient tool to measure the effectiveness of novel radiation equipment for therapy via a combination with a phantom model.
